# Accumulation of Securin on Spindle During Female Meiosis I

**DOI:** 10.3389/fcell.2021.701179

**Published:** 2021-07-29

**Authors:** Tereza Pauerova, Lenka Radonova, Adela Horakova, Jason G. Knott, Martin Anger

**Affiliations:** ^1^Department of Genetics and Reproduction, Veterinary Research Institute, Brno, Czechia; ^2^Department of Experimental Biology, Faculty of Science, Masaryk University, Brno, Czechia; ^3^Department of Animal Science, Michigan State University, East Lansing, MI, United States

**Keywords:** securin, separase, oocyte, mouse, meiosis

## Abstract

Chromosome segregation during female meiosis is frequently incorrect with severe consequences including termination of further development or severe disorders, such as Down syndrome. Accurate chromosome segregation requires tight control of a protease called separase, which facilitates the separation of sister chromatids by cohesin cleavage. There are several control mechanisms in place, including the binding of specific protein inhibitor securin, phosphorylation by cyclin-dependent kinase 1 (CDK1), and complex with SGO2 and MAD2 proteins. All these mechanisms restrict the activation of separase for the time when all chromosomes are properly attached to the spindle. In our study, we focused on securin and compared the expression profile of endogenous protein with exogenous securin, which is widely used to study chromosome segregation. We also compared the dynamics of securin proteolysis in meiosis I and meiosis II. Our study revealed that the expression of both endogenous and exogenous securin in oocytes is compartmentalized and that this protein accumulates on the spindle during meiosis I. We believe that this might have a direct impact on the regulation of separase activity in the vicinity of the chromosomes.

## Introduction

In mitosis, securin inhibits separase until all chromosomes are attached properly to the spindle, and their sister kinetochores are bi-oriented facing the opposite spindle poles (Ciosk et al., 1998; Salah and Nasmyth, 2000; Meadows and Millar, 2015). When this is achieved, spindle assembly checkpoint (SAC) turns off, which leads to the activation of anaphase promoting complex/cyclosome (APC/C) and consequently to the initiation of securin proteolysis (Pines, 2011). In some species, for example, in fission yeast, securin is essential for correct chromosome segregation at anaphase (Uzawa et al., 1990). In budding yeast, mutations in Pds1 limit growth capacity, and this effect is temperature-dependent and manifested by chromosome segregations errors and spindle defects (Yamamoto et al., 1996). In mouse and human tissue culture cells, securin seems to be dispensable for the fidelity of chromosome segregation (Yamamoto et al., 1996; Mei et al., 2001; Pfleghaar et al., 2005). However, even in these cells, securin is vital for correct timing of anaphase entry (Mei et al., 2001).

Similar to mitosis, the ubiquitination of securin during mammalian female meiosis I is facilitated by APC/C and initiated only after silencing of SAC (McGuinness et al., 2009). In mouse oocytes, the depletion of securin is not sufficient for separase premature activation, which emphasizes the role of maturation promoting factor (MPF) in the control of separase activity in these cells (Thomas et al., 2019). However, in case of separase resistance to MPF phosphorylation, the securin becomes essential and sufficient for accurate timing of separase activation. It seems, therefore, that during meiosis I, the securin and cyclin-dependent kinase 1 (CDK1) both play important, albeit redundant, roles in controlling the separase activity. Both mechanisms together ensure that chromosome segregation, vital for cell survival, will not start prematurely. The role of securin is perhaps even more important in oocytes undergoing meiosis II. At this stage, mammalian oocytes are arrested by the activity of the cytostatic factor (CSF), whose function is to prevent anaphase entry by inhibiting APC/C activation and consequently decline of CDK1 activity (Madgwick and Jones, 2007). It was reported that the depletion of securin in meiosis II, in contrast to the inhibition of CDK1, is sufficient for the initiation of premature separation of sister chromatids (Nabti et al., 2008). Maintaining the stable securin levels in meiosis II is, therefore, essential in situations such as oocyte aging, during which the securin reduction, caused by unscheduled APC/C activation, might lead into the precocious separation of sister chromatids (Nabti et al., 2017).

In this study, we assessed the dynamics of securin expression during meiosis I and II. In particular, we present a quantitative comparison of securin levels during meiosis I and meiosis II, focusing on the expression of endogenous protein as well as securin administrated by microinjection. Our data also revealed that the overexpression increases securin levels only moderately. We compared the dynamics of securin destruction between meiosis I and meiosis II, demonstrating that this process is significantly faster in meiosis II. Lastly, the analysis of fixed samples as well as data from live cell imaging showed that the securin expression is compartmentalized during meiosis I, with the accumulation of the protein on spindle. We believe that the accumulation of securin in the spindle area might be important for its function in the inhibition of separase and in the protection of the cohesion between chromosomes.

## Results

### The Expression and Proteolysis of Endogenous and Exogenous Securin During Meiosis I

**FIGURE 1:**
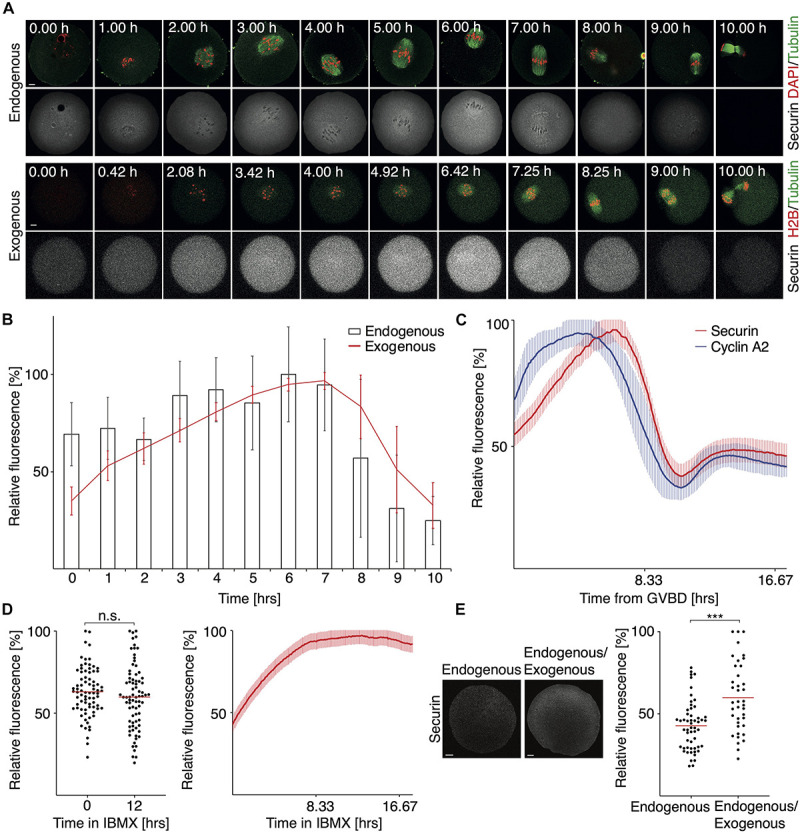
The expression and proteolysis of endogenous and exogenous securin during meiosis I. **(A)** Upper panel shows representative images of fixed oocytes from indicated time intervals after IBMX removal. DNA (red) was visualized by DAPI, tubulin (green) and securin (gray) were detected by anti-tubulin and anti-securin antibodies. Scale bar represents 10 μm. Lower panel shows representative time frames from live cell imaging of oocytes microinjected with cRNAs encoding histone H2B (red), tubulin (green), and securin (gray) fused to fluorescent proteins. Scale bar represents 20 μm. **(B)** Expression profiles of endogenous securin (columns, 0 h *n* = 26, 1 h *n* = 25, 2 h *n* = 26, 3 h *n* = 26, 4 h *n* = 26, 5 h *n* = 26, 6 h *n* = 26, 7 h *n* = 26, 8 h *n* = 25, 9 h *n* = 21, and 10 h *n* = 24) and exogenous securin (red line, all time intervals *n* = 37). Time from IBMX removal. The data for endogenous securin were obtained from two independent experiments and for exogenous securin from three independent experiments. **(C)** Fluorescence signal profiles of securin (red, *n* = 28) and cyclin A2 (blue, *n* = 28) during meiosis I. Time is from GVBD. The data were obtained from three independent experiments. **(D)** Left panel shows scatterplot of the endogenous securin signal in oocytes at 0 h (mean: 63.01%, *n* = 78) and at 12 h (mean: 59.84%, *n* = 78) of IBMX treatment. The difference between groups was not statistically significant (α > 0.05, n.s. *P* = 0.2554). The data were obtained from three independent experiments. Right panel shows fluorescence signal levels of exogenous securin (red, *n* = 28) in oocytes cultured in media with IBMX for 18 h (GV arrested oocytes). The data were obtained from three independent experiments. **(E)** The left part shows detection of securin by antibody in non-injected and injected MI oocytes. Both groups of oocytes were harvested 4 h after GVBD and synchronized in MG132, before securin detection. Scale bar represents 10 μm. The scatterplot shows non-injected oocytes (endogenous; mean: 42.76%, *n* = 55) and injected (endogenous/exogenous; mean: 59.88%, *n* = 39) MI oocytes. The difference between groups was statistically significant (α < 0.05, ***P = 0.0001). The data were obtained from three independent experiments.

The dynamics of securin expression during meiosis I was studied mostly using exogenous securin (Kudo et al., 2006; McGuinness et al., 2009). However, the information about how precisely the expression of exogenous securin recapitulates its endogenous counterpart, obtained by similar method and in comparable time intervals, is missing. Here we used fluorescence microscopy and measured the relative expression levels of both during meiosis I (Figure 1A). Our results showed that the relative expression profiles of exogenous and endogenous proteins are indeed very similar (Figure 1B and Supplementary Figure 1A). After the initial accumulation, the securin expression peaked around 6–7 h, after which we observed a sharp decline, as cells approached anaphase.

The securin decrease was observed for the next 2–3 h, and it was followed by chromosome segregation, which could be observed in the case of exogenous securin in live cell imaging experiments. Importantly, the dynamics of the securin signal loss during the metaphase to anaphase transition was similar in the case of endogenous as well as exogenous securin. This indicates that both proteins are targeted by APC/C, as it was previously shown for exogenous securin (McGuinness et al., 2009), with similar efficiency and dynamics. In contrast to this, when we compared the proteolysis of securin and cyclin A2, the dynamics of both events was quite different (Figure 1C). Although the cyclin A2 proteolysis starts before SAC is turned off (Karasu et al., 2019) and the securin destruction starts after SAC inactivation, the different dynamics of the proteolysis of both proteins caused that the proteins disappeared from cells at similar time.

In our experiments, we, however, noticed a difference in the behavior of the endogenous and exogenous protein. If oocytes with intact nucleus (GV stage oocytes) were cultured in the presence of isobutylmethylxanthine (IBMX, inhibitor of meiotic resumption) for a prolonged period of time, the endogenous securin remained constant, whereas the level of microinjected securin increased significantly during this time (Figure 1D). This could indicate a UTR-based regulation of translation, since the securin-specific UTRs are not present in microinjected securin ORF.

Although the results presented in Figures 1A,B allowed the time-resolved comparison of securin levels during meiosis I, the data were obtained by the combination of antibody detection and fluorescent protein tagging, which does not allow a direct comparison. Therefore, we scored total securin level by antibody detection in injected and non-injected oocytes, synchronized in metaphase I by the 26S proteasome inhibitor MG132 (Figure 1E). Our data showed that in cells injected by exogenous securin, the signal was higher on average by 18% in comparison to the control cells. We obtained similar data with unsynchronized cells indicating that the levels of securin were not affected by MG132 synchronization (Supplementary Figure 1C).

### The Dynamics of Securin Expression During Meiosis II

**FIGURE 2:**
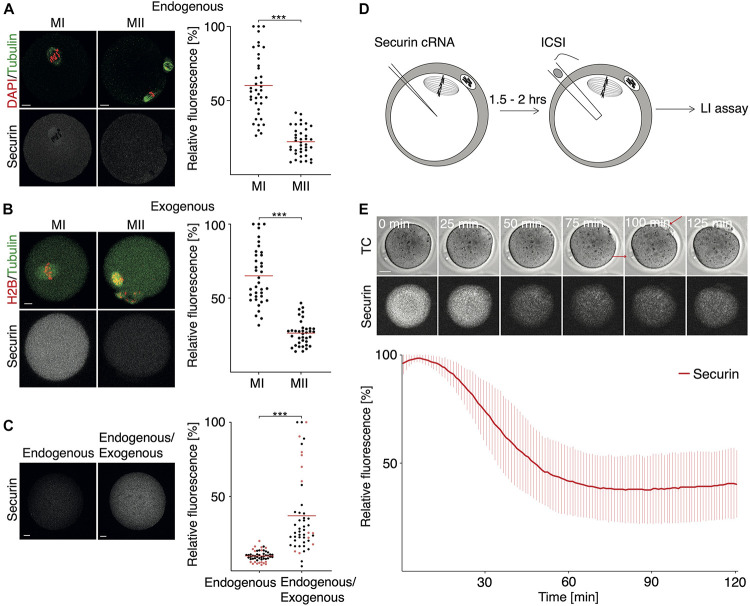
The dynamics of securin expression during meiosis II. **(A)** Representative images of fixed MI and MII oocytes and endogenous securin detection. DNA (red) is visualized by DAPI. Tubulin (green) and securin (gray) were detected by anti-tubulin and anti-securin antibodies. Scale bar represents 10 μm. The scatterplot shows relative signal of endogenous securin in meiosis I (MI; mean: 60.18%; *n* = 39) and meiosis II (MII; mean: 22.16%, *n* = 38). The difference between MI and MII oocytes was statistically significant (α < 0.05, ***P < 0.0001). The data were obtained from three independent experiments. **(B)** Representative images of meiosis I (MI) and meiosis II (MII) oocytes from live cell imaging experiment. Oocytes were microinjected with cRNA encoding histone H2B (red), tubulin (green), and securin (gray) fused to fluorescent proteins. Scale bar represents 20 μm. The scatterplot shows relative numbers representing maximum securin expression level in each individual cell in meiosis I (MI; mean: 65.05%, *n* = 37) and meiosis II (MII; mean: 26.36%, *n* = 37). The difference between groups was statistically significant (α < 0.05, ***P < 0.0001). The data were obtained from three independent experiments (the data shown here are from the same dataset as in Figure 1B exogenous). **(C)** Representative images of fixed MII oocyte (left, endogenous) and MII oocyte injected with cRNA encoding securin (right, endogenous/exogenous); securin is detected by antibody in both. Injected oocytes were fixed 2 h (black dots) or 5 h (red dots) after microinjection. Scale bar represents 10 μm. The scatterplot shows relative securin signal in non-injected (endogenous; mean: 10.04%, *n* = 51) or injected (endogenous/exogenous; mean: 37.08%, *n* = 50) MII oocytes. The difference between groups was statistically significant (α < 0.05, ***P < 0.0001). The data were obtained from three independent experiments. **(D)** Overview of experimental setup. *In vivo* MII oocytes were microinjected with cRNA encoding securin fused to fluorescent protein. MII oocytes were then activated by ICSI and subsequently monitored by time-lapse confocal microscopy. The live cell imaging started within 10 min after ICSI. **(E)** Upper part shows representative time frames from **(D)** showing the fluorescence signal of securin (gray) in MII oocyte after ICSI. Red arrows mark first and second polar body. Scale bar represents 20 μm. Lower part shows relative securin signal after ICSI (red line, *n* = 11). The data were obtained from four independent experiments.

It was shown previously that the securin expression driven by microinjected cRNA is lower in meiosis II in comparison to meiosis I (Kudo et al., 2006; Nabti et al., 2008; McGuinness et al., 2009). This seems to be similar also in the case of the endogenous protein (Nabti et al., 2008); however, the precise quantification of the difference between meiosis I and II is not available. Here we compared the endogenous levels of securin in meiosis I oocytes and metaphase II eggs (Figure 2A). The results showed that the average securin levels are 3–3.7 times lower in meiosis II compared to meiosis I in endogenous as well as exogenous protein (Figure 2B).

Since transcription is silenced during meiosis and the securin protein is destroyed during metaphase I to anaphase I transition, the expression of securin in meiosis II is perhaps driven by the remaining pool of mRNA left after translation in meiosis I. To assess whether the levels of securin in meiosis II are lower due to a specific regulation or due to the lack of mRNA, we microinjected securin cRNA into metaphase II oocytes and measured the total securin protein in control and injected cells by immunofluorescence (Figure 2C). Our data showed that the administration of cRNA at this stage increased significantly the securin protein levels. The securin levels remained higher even after prolonged incubation of metaphase II oocytes for 5 h (Figure 2C – red dots).

In order to measure the dynamics of securin proteolysis in meiosis II, we used intracytoplasmic sperm injection (ICSI) to overcome the meiosis II arrest and to trigger the continuation of the cell cycle (Figure 2D). Our results showed that in comparison to meiosis I, the destruction of securin in meiosis II is significantly faster (Figure 2E), which is in accordance with previously published results (Chang et al., 2004). Whereas after APC/C activation in meiosis I the securin proteolysis takes approximately 2–3 h (Figure 1B), in meiosis II, the same process is completed within approximately 1 h (Figure 2E), and, as in meiosis I, the destruction of securin is followed by the separation of chromosomal masses (anaphase—data not shown).

### Securin Accumulates on Meiosis I Spindle

**FIGURE 3:**
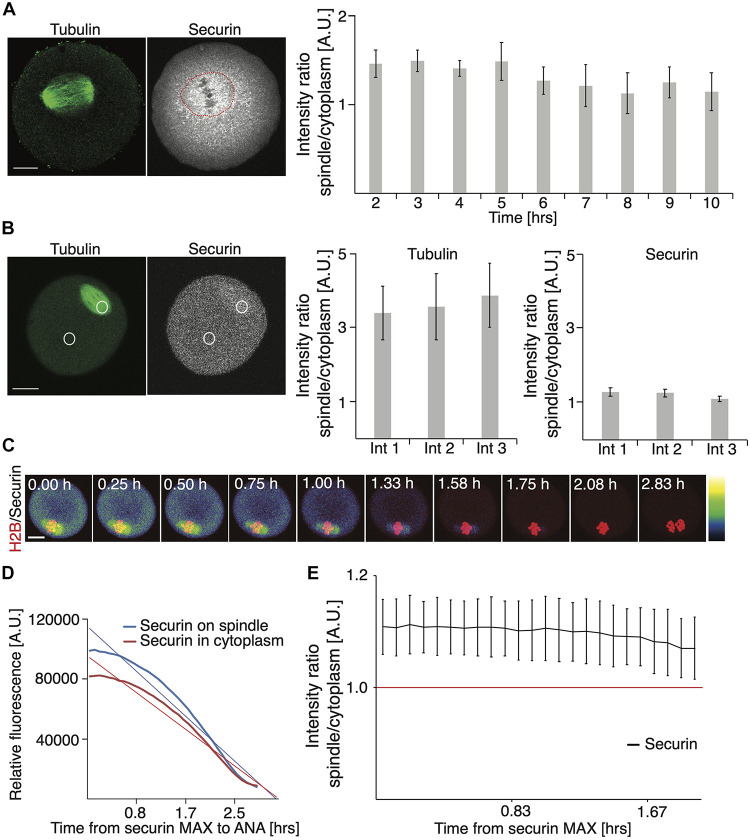
Securin accumulates on meiosis I spindle. **(A)** Representative image of fixed oocyte showing endogenous tubulin (green) and securin (gray) proteins detected by antibodies. Scale bar represents 20 μm. The red circle indicates the position of the spindle. Graph on right shows the ratio between securin signal on the spindle and in the whole cell 2 h (1.45; *n* = 22), 3 h (1.49; *n* = 25), 4 h (1.40; *n* = 23), 5 h (1.48; *n* = 26), 6 h (1.26; *n* = 26), 7 h (1.21; *n* = 26), 8 h (1.12; *n* = 24), 9 h (1.24; *n* = 20), and 10 h (1.14; *n* = 18) after release from IMBX. The data were obtained from two independent experiments (the data shown here are from the same dataset as in Figure 1B endogenous). **(B)** Example of individual oocyte and regions of interest used for analysis of proportion of securin and tubulin on the spindle and in cytoplasm. Cells were injected with cRNA encoding securin and tubulin fused to fluorescent proteins. Scale bar represents 20 μm. The left graph shows the ratio of tubulin fluorescence signal on spindle and in cytoplasm in three intervals: 1 (ratio 3.39, *n* = 18), 2 (ratio 3.56; *n* = 18), and 3 (ratio 3.86; *n* = 18). The right graph shows the ratio of exogenous securin fluorescence signal on spindle and in cytoplasm in three intervals: 1 (ratio 1.29, *n* = 18), 2 (ratio 1.26; *n* = 18), and 3 (ratio 1.11; *n* = 18). Intervals were selected individually for each cells and represent the time of securin maximum (1), the time in the middle between interval 1 and 3 (2), and 20 min before anaphase (3). The data were obtained from four independent experiments. **(C)** Representative time frames from live cell imaging of oocytes injected with cRNA encoding securin and histone H2B fused to fluorescent proteins. The securin levels are indicated by color grading from lowest (black) to highest (white) and the time starts at securin maximum. Scale bar represents 20 μm. **(D)** Graph shows exogenous securin levels on spindle (blue) and in cytoplasm (red) in individual oocyte. Data are shown from securin maximum to anaphase. The corresponding linear trend lines are shown. **(E)** Graph shows the ratio of exogenous securin signal on spindle and in cytoplasm in oocytes after injection of cRNA encoding securin fused to fluorescent protein (*n* = 13). Time starts from securin maximum. The data were obtained from three independent experiments.

In our securin expression experiments, we noticed that the securin protein accumulated on the meiosis I spindle (Figure 1A), and in the cytoplasm, the signal was lower. Such an expression pattern was previously reported in fission yeast (Kumada et al., 1998), in HeLa cells (Hagting et al., 2002), and in budding yeasts, where Pds1 is also required for the localization of separase to the spindle (Jensen et al., 2001). To analyze this phenomenon quantitatively, we focused first on endogenous protein and measured a ratio between the signal localized within the area of the spindle and the signal in the whole cell (Figure 3A and Supplementary Figures 1B,E). Our data showed that during meiosis I, since the spindle formation until very close to anaphase, this ratio was always in favor of the spindle (Figure 3A and Supplementary Figure 1B).

To test whether this is not caused by an artifact of fixation and sample preparation, during which the soluble proteins might be lost, we measured a similar ratio in live cells, microinjected with cRNA encoding fluorescently tagged tubulin and securin (Figure 3B). In three time intervals, selected with respect to securin expression in each individual cell, namely, at the highest securin expression before APC/C activation, at the middle of APC/C activity, and 20 min prior to anaphase, we measured the ratio of tubulin and securin on the spindle and in the cytoplasm. Our data showed that although this ratio was not as high as in the case of tubulin, in all three intervals, overexpressed securin was accumulated significantly more on the spindle (Figure 3B).

In order to exclude a possibility of interaction of overexpressed tubulin and securin, we quantified the ratio between the spindle localized and the cytoplasmic fraction of securin in cells without tubulin overexpression. Cells were microinjected with the cRNA of securin and histone only, and the distribution of securin on the spindle and in the cytoplasm was monitored by live cell imaging (Figure 3C). Our results showed that during the time intervals following the highest securin expression, the securin signal was consistently higher on the spindle (Figures 3C–E). The results also show that the signal on the spindle is being reduced more rapidly due to its higher initial value (Figure 3D). Importantly, in the intervals following maximal securin expression, the average ratio between the securin signal on the spindle and in the cytoplasm was always in favor of the spindle (Figure 3E).

## Discussion

The expression of exogenous securin, driven from a transgene or from microinjected RNA, is widely used for live cell imaging studies. Our results demonstrated that during meiosis I, the exogenous securin showed a very similar expression pattern to the endogenous protein, including the timing and the dynamics of its proteolysis. We also show here that overexpression after microinjection elevates securin levels in oocytes only moderately, on average by 20%. Our experiments, however, revealed an important difference related to the expression of securin in GV stage oocytes. Unlike the endogenous protein, securin translated from microinjected cRNA exhibited significant accumulation over time (Figure 1D). This perhaps suggests that the regulation of translation is facilitated by the securin-specific UTRs, which are absent in microinjected cRNA. The regulation of expression levels by UTRs during meiosis II was previously reported in the case of Sgo1 in *Schizosaccharomyces pombe* (Rabitsch et al., 2004; Gregan et al., 2008). It is also known that certain mRNAs, including MOS and cyclin B1, are being recruited for translation in oocytes by binding of cytoplasmic polyadenylation element binding protein 1 (CPEB1) (Richter, 2007; Meneau et al., 2020). For example, the translation of cyclin B1 in mouse oocytes starts approximately 2 h after GVBD (Han et al., 2017), and the expression is therefore low in GV oocytes, similarly to securin. However, we were unable to identify a conserved sequence in securin UTRs, which is recognized by CPEB1. The expression levels of cyclin B1 are controlled in part by APC/C at the GV stage (Reis et al., 2006). The expression pattern of exogenous securin would, however, suggest that the protein is perhaps not targeted by APC/C at this stage, since its levels after 12 h doubled. Importantly, both endogenous and exogenous proteins show similar dynamics of proteolysis, which starts around 6–7 h after GVBD. This indicates that APC/C, which was shown to be responsible for the targeting of exogenous protein (McGuinness et al., 2009), ubiquitinates both proteins with similar efficiency. Although securin proteolysis takes 2–3 h (Figure 1B), it is more efficient than proteolysis of cyclin A2 overexpressed in the same cell (Figure 1C). Although cyclin A2 destruction is initiated prior to securin, both proteins reach their lowest level at similar time.

The securin protein levels are significantly lower in meiosis II than in meiosis I (Figures 2A,B), and this difference was similar for both endogenous and exogenous proteins, on average 3.7 and 3 times less in meiosis II, respectively. This is interesting and the excess of securin in meiosis I might have a specific role. It is conceivable that there is also an excess of separase in meiosis I, and then the extra securin is required to equilibrate this. It is also possible that securin is required as a substrate of APC/C in meiosis I, as a part of APC/C activity control (Thomas et al., 2019). Administration of fresh cRNA into meiosis II oocytes increases the securin levels on average three times (Figure 2C), indicating that the lower expression of securin in meiosis II might be due to a lack of mRNA rather than subject of specific regulation. Securin proteolysis in meiosis I takes about 2–3 h (Kudo et al., 2006; McGuinness et al., 2009; and results here), whereas in meiosis II, the securin is reduced much faster during approximately 1 h (Chang et al., 2004; Madgwick et al., 2004; and the results here). This difference reflects the amount of protein, which differs 3–3.7 times.

We show here that, in oocyte meiosis I, securin is significantly more accumulated on the spindle. The accumulation of securin on the spindle was shown before in yeasts mitotic and meiotic cells (Kumada et al., 1998; Jensen et al., 2001; Kitajima et al., 2003) and, also without further details, in mammalian cells (Hagting et al., 2002). The dynamics of securin in the vicinity of the chromosomes was recently systematically studied in HeLa cells (Konishi et al., 2018), and it was shown that securin levels and degradation speed differ between this population and cytoplasmic securin. In our case, the accumulation on the spindle was detected in the case of not only exogenously expressed protein but also endogenous securin (Figure 3; Kumada et al., 1998; Hagting et al., 2002). Whereas the exogenous protein seems to be located uniformly within the area of the spindle, the endogenous protein shows spots concentrated in the vicinity of the chromosomes (Figure 1A and Figure 3A). The ratio between the spindle and the cytoplasm population of securin seems to be highest before the activation of APC/C (Figure 3). Nevertheless, the difference between the securin concentration on the spindle and in the cytoplasm is preserved almost until anaphase (Figures 3C–E). It is conceivable that the accumulation of securin on the spindle, in the vicinity of the chromosomes, is functionally important for timely restriction of separase activity. It was shown that securin is required for separase localization to the spindle (Kumada et al., 1998; Jensen et al., 2001). Also measuring the separase activity by a FRET sensor showed that the separase is activated in oocyte meiosis I tens of minutes before anaphase (Nikalayevich et al., 2018; Karasu et al., 2019; Thomas et al., 2019), which is in accordance with the loss of the securin signal on the spindle observed in our experiments. To evaluate whether the accumulation of securin on the spindle has functional significance will require additional experimental work. Also, the mechanism of securin accumulation on the spindle is unknown and remains to be discovered.

## Materials and Methods

### Animals

All animal work was conducted according to Act No 246/1992 Coll. on the protection of animals against cruelty. It was approved by the Central Commission for Animal Welfare, approval ID 51/2015. At least 12-week-old CD-1 females (Animal Breeding and Experimental Facility, Faculty of Medicine, Masaryk University) were used for all experiments.

### Oocyte Isolation

GV oocytes were isolated by a technique described previously (Radonova et al., 2020). For MII oocytes isolation, mice were stimulated with pregnant mare serum gonadotropin (PMSG, 5 IU, Merck) followed 44–48 h by next stimulation with human chorionic gonadotropin (hCG, 5IU, Merck). MII oocytes were isolated 16–17 h later by manual rupturing of oviduct in the M2 medium (Merck) with 0.05% hyaluronidase (Merck). MII oocytes were then cultivated in the KSOM + AA medium (Caisson Laboratories) under the mineral oil (NidOil) in standard conditions (37°C, 5% CO_2_).

### Microinjection, Live Cell Imaging Assay

Germinal vesicle (GV) oocytes were microinjected by a technique described previously (Radonova et al., 2020). MII oocytes were microinjected in an M2 medium (Merck) supplemented with 2.5% sucrose (JT Baker). The following cRNAs fused with fluorescent proteins were used: securin-CFP, securin-EGFP, tubulin-Venus, histone H2B-Venus, cyclin A2-mCherry, and histone H2B-mCherry.

LI assay was performed on the Leica SP5 and Olympus FluoView 3000 microscopes with an EMBL incubator (37°C, 5% CO_2_). Oocytes were scanned using HCX PL APO 40×/1.1 water objective, UPLSAPO 30×/1.05, and UPLSAPO 40×/1.406 silicone objective. The following wavelengths were used for the detection of CFP, EGFP, Venus, and mCherry fluorescent signal: 445, 458, 488, 514, 561, and 594 nm.

### Sperm Preparation, ICSI

Intracytoplasmic sperm injection and sperm collection were performed as previously described (Bernhardt et al., 2018) with the following modifications. B6D2F1 male mouse was used for sperm collection. Swim-out was done in a 500-μl drop of an M16 medium (Merck) for 15 min. ICSI was performed by the injection of a single sperm head into a previously microinjected MII egg with cRNA encoding fluorescently tagged securin and histone H2B. Micromanipulation was done on a Leica DMI3000 B inverted microscope equipped with InjectMan NI 2 and TransferMan NK 2 micromanipulators (Eppendorf), Piezo PMM 4G (Prime tech), and an IM-11-2 pneumatic microinjector (Narishige). Live cell assay was performed as previously described within 10 min after the group of eggs was injected by sperm. Eggs were scanned with a resonant scanner every minute for 2 h after activation. HCX PL APO 40×/1.1 water CS and/or UPLSAPO 30× S/1.05 silicone objectives were used.

### Immunostaining

In experiments showing the dynamics of endogenous securin, the GVs were cultured in an M16 medium (Merck); every hour, a group of cells was removed and fixed, and target proteins were detected by primary antibodies with subsequent visualization by secondary antibodies fused to fluorescent tags.

In experiments comparing exogenous/endogenous securin levels in MI and MII oocytes, in the case of MI, the GVs were firstly microinjected and matured until MI (5.5–6 h), then were fixed and proceeded to immunostaining. In two experiments, MI cells were synchronized for 3 h in 10 μM MG132 (Merck) before immunostaining. In the case of MII, the MII eggs were firstly microinjected, cultured for 2 or 5 h for expression of injected cRNA, fixed, and proceeded to immunostaining. In both cases, control cells were simultaneously prepared, with the exclusion of microinjection.

In all cells, *zona pellucida* was removed by briefly incubating in Tyrode’s solution (Merck) and then fixed in 3.7% paraformaldehyde (Merck) for 1 h. Then oocytes were permeabilized with 0.1% Triton X-100 (Merck) for 15 min and blocked (0.1% BSA) for 1 h. The following antibodies were used: securin (1:250 dilution, Thermo Fisher Scientific, catalog # 700791), anti-acetylated tubulin (1:500 dilution, Merck), Alexa Fluor 488 goat anti-rabbit (1:500 dilution, Thermo Fisher Scientific), Alexa Fluor 647 goat anti-mouse (1:500 dilution, Thermo Fisher Scientific), and Alexa Fluor 647 goat anti-rabbit (1:500 dilution, Thermo Fisher Scientific). All cells were mounted in vectashield with DAPI (Vector Laboratories). Fixed oocytes were scanned on Leica SP5 and an Olympus FluoView 3000 confocal microscope equipped with an HCS PL APO 63×/1.4 oil objective and UPLSAPO 60×/1.406 silicone objective. The following wavelengths were used for the detection of DAPI, Alexa Fluor 488, and Alexa Fluor 647 signals: 405, 488, 633, or 640 nm.

### Image and Statistical Analysis

All obtained data were processed and analyzed using the Imaris software 9.2.1, 9.5.0^1^ and ImageJ 1.52m (National Institutes of Health). For statistical analysis, the GraphPad Prism 9 for macOS software was used with the following tests: D’Agostino and Pearson omnibus normality test, Student’s *t*-test, and Mann-Whitney *U* test.

## Data Availability Statement

The original contributions presented in the study are included in the article/Supplementary Material, further inquiries can be directed to the corresponding author.

## Ethics Statement

All animal work was conducted according to Act No 246/1992 Coll. on the protection of animals against cruelty. It was approved by the Central Commission for Animal Welfare, approval ID 51/2015.

## Author Contributions

MA: conceptualization, supervision, and funding. TP, LR, and AH: experimental work, data acquisition, and data analysis. JK: enabling appropriate methods. MA, TP, and LR: writing. All authors contributed to the article and approved the submitted version.

## Conflict of Interest

The authors declare that the research was conducted in the absence of any commercial or financial relationships that could be construed as a potential conflict of interest.

## Publisher’s Note

All claims expressed in this article are solely those of the authors and do not necessarily represent those of their affiliated organizations, or those of the publisher, the editors and the reviewers. Any product that may be evaluated in this article, or claim that may be made by its manufacturer, is not guaranteed or endorsed by the publisher.

## Footnotes

1
www.bitplane.com

## Supplementary Material

Supplementary Figure 1 **(A)** Graph shows individual experiments used in Figure 1B. **(B)** Graph shows individual experiments used in Figure 3A. **(C)** The scatterplot shows relative securin signal in non-injected (endogenous; mean: 37.24%; *n* = 15) and injected (endogenous/exogenous; mean: 51.19%; *n* = 17) MI oocytes. GV oocytes were injected with cRNA encoding securin or uninjected, matured and harvested 4 h after GVBD, then processed for immunodetection for securin. The difference between groups was statistically significant (α < 0.05; * *P* = 0.0465). The data were obtained from one experiment. **(D)** The scatterplot shows level of injected cRNA of securin in MII oocytes (exogenous; mean: 58.59%, *n* = 14) before fixation and immunodetection as showed in Figure 2C. The same dataset was used as in Figure 2C with prolonged time to 5 h before cell fixation. **(E)** Example of analysis of securin level in fixed oocytes used for Figure 3A. The white circles indicate region of interest used for fluorescence signal intensity measurement on spindle (upper panel) and in whole cell (lower panel). Scale bar represents 20 μm.
