# Supplementary figures and images for: Accumulation of Securin on Spindle During Female Meiosis I
Source: Front Cell Dev Biol. 2021 Jul 29;9:701179. doi: 10.3389/fcell.2021.701179 (PMC8358270; doi:10.3389/fcell.2021.701179)

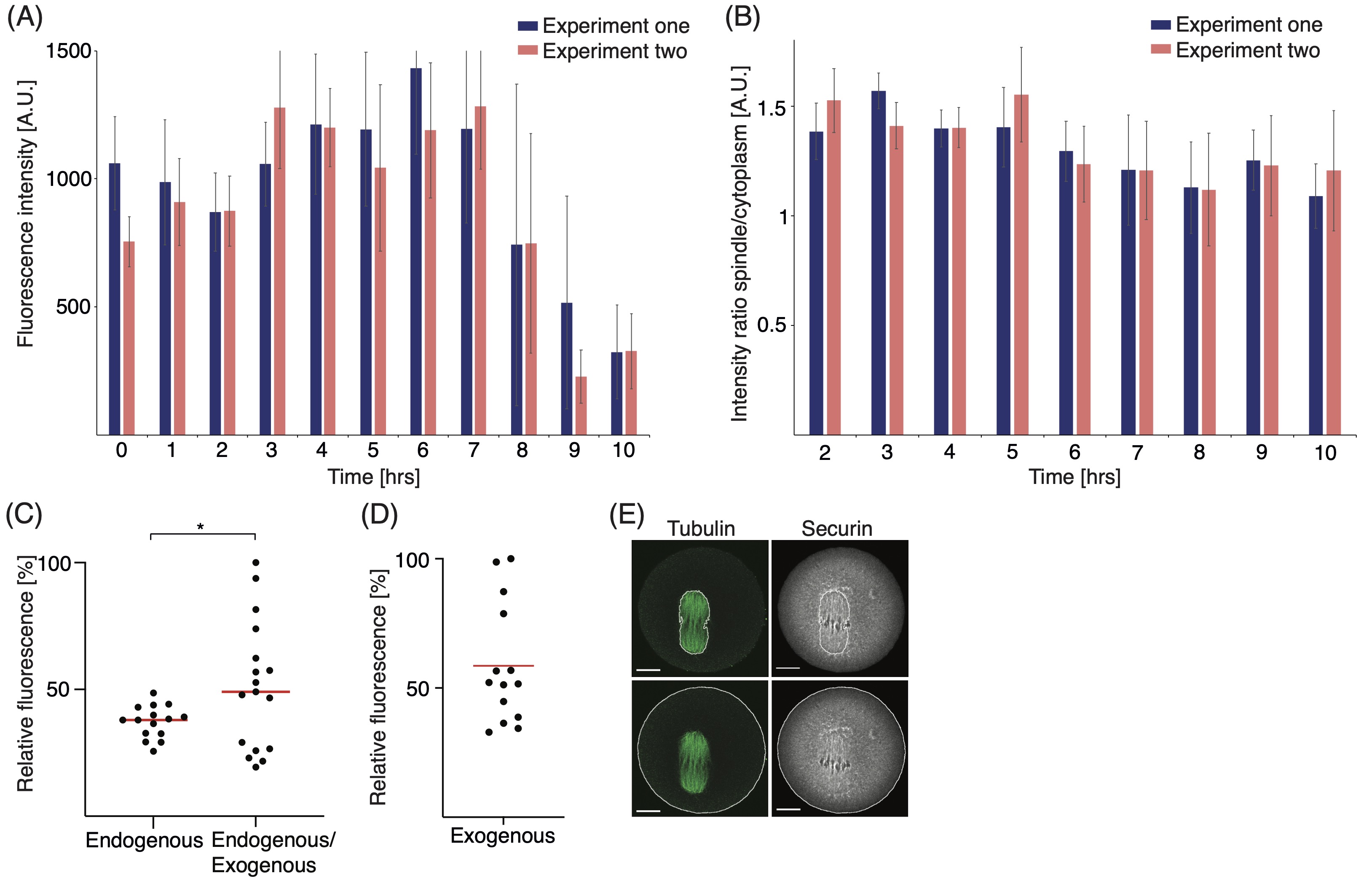

Supplement: Supplementary Figure 1 — (A) Graph shows individual experiments used in Figure 1B. (B) Graph shows individual experiments used in Figure 3A. (C) The scatterplot shows relative securin signal in non-injected (endogenous; mean: 37.24%; n = 15) and injected (endogenous/exogenous; mean: 51.19%; n = 17) MI oocytes. GV oocytes were injected with cRNA encoding securin or uninjected, matured and harvested 4 h after GVBD, then processed for immunodetection for securin. The difference between groups was statistically significant (α < 0.05; * P = 0.0465). The data were obtained from one experiment. (D) The scatterplot shows level of injected cRNA of securin in MII oocytes (exogenous; mean: 58.59%, n = 14) before fixation and immunodetection as showed in Figure 2C. The same dataset was used as in Figure 2C with prolonged time to 5 h before cell fixation. (E) Example of analysis of securin level in fixed oocytes used for Figure 3A. The white circles indicate region of interest used for fluorescence signal intensity measurement on spindle (upper panel) and in whole cell (lower panel). Scale bar represents 20 μm. [file Image_1.JPEG]
